# Comparative Effectiveness of Levothyroxine, Desiccated Thyroid Extract, and Levothyroxine+Liothyronine in Hypothyroidism

**DOI:** 10.1210/clinem/dgab478

**Published:** 2021-06-29

**Authors:** Mohamed K M Shakir, Daniel I Brooks, Elizabeth A McAninch, Tatiana L Fonseca, Vinh Q Mai, Antonio C Bianco, Thanh D Hoang

**Affiliations:** 1 Walter Reed National Military Medical Center, Bethesda, MD 20889-5600, USA; 2 Uniformed Services University of the Health Sciences, Bethesda, MD 20814, USA; 3 Division of Endocrinology and Metabolism, Rush University Medical Center, Chicago, IL 60612, USA; 4 Section of Adult and Pediatric Endocrinology, University of Chicago, Chicago, IL 60637, USA

**Keywords:** liothyronine, levothyroxine, desiccated thyroid extract, hypothyroidism, quality of life

## Abstract

**Introduction:**

Studies comparing levothyroxine (LT4) therapy with LT4 + liothyronine (LT3) or desiccated thyroid extract (DTE) did not detect consistent superiority of either treatment. Here, we investigated these therapies, focusing on the whole group of LT4-treated hypothyroid patients, while also exploring the most symptomatic patients.

**Methodology:**

Prospective, randomized, double-blind, crossover study of 75 hypothyroid patients randomly allocated to 1 of 3 treatment arms, LT4, LT4 + LT3, and DTE, for 22 weeks. The primary outcomes were posttreatment scores on the 36-point thyroid symptom questionnaire (TSQ-36), 12-point quality of life general health questionnaire (GHQ-12), the Wechsler memory scale-version IV (VMS-IV), and the Beck Depression Inventory (BDI). Secondary endpoints included treatment preference, biochemical and metabolic parameters, etiology of hypothyroidism, and Thr92Ala-DIO2 gene polymorphism. Analyses were performed with a linear mixed model using subject as a random factor and group as a fixed effect.

**Results:**

Serum TSH remained within reference range across all treatment arms. There were no differences for primary and secondary outcomes, except for a minor increase in heart rate caused by DTE. Treatment preference was not different and there were no interferences of the etiology of hypothyroidism or Thr92Ala-DIO2 gene polymorphism in the outcomes. Subgroup analyses of the 1/3 most symptomatic patients on LT4 revealed strong preference for treatment containing T3, which improved performance on TSQ-36, GHQ-12, BDI, and visual memory index (VMS-IV component).

**Conclusions:**

As a group, outcomes were similar among hypothyroid patients taking DTE vs LT4 + T3 vs LT4. However, those patients that were most symptomatic on LT4 preferred and responded positively to therapy with LT4 + LT3 or DTE.

Hypothyroidism, or underactive thyroid function, is a common condition characterized by cognitive and metabolic impairments (1). It is estimated that 3.7% of the general US population is affected by hypothyroidism based on The National Health and Nutrition Examination Survey (1999-2002) (2).

Various forms of thyroid extracts were originally developed in the 1880s, with desiccated thyroid extract (DTE) becoming the dominant form and commercially available in the early 1900s. During the 1970s, there was a switch to therapy with levothyroxine (LT4), which to this day is the standard of care, considered safe and effective (3). The switch occurred rapidly, but some anecdotal evidence emerged that a few patients did not feel well on LT4 and asked to return to DTE (4). The existence of residual symptoms in LT4-treated patients was first documented through a patient survey (5) and later through questionnaires that evaluated thyroid-specific symptoms and quality of life (QoL) (6), as well as sophisticated cognitive tests (7). Several clinical trials addressed the question of whether residual symptoms could be resolved through the use of combination therapy with LT4 and liothyronine (LT3), but evidence of consistent superiority of combination therapy was not obtained (8,9). A case could be made that, in some instances, patients prefer the combination approach (10,11). Indeed, in a recent randomized, double-blind, crossover study (12), we confirmed that QoL of hypothyroid patients was similarly improved with LT4 or DTE, but the latter was associated with modest weight loss (~4 pounds); nearly 50% of the study patients preferred treatment with DTE over LT4.

Most professional medical guidelines (3) recommend LT4 monotherapy “as the preparation of choice” for thyroid hormone replacement, and against the “routine use” of combination treatment with LT4 and LT3 or DTE because of uncertainty about long term efficacy and potential safety concerns. A recent consensus statement by the American, European, and British Thyroid Associations reasoned that previous clinical trials did not focus sufficiently on patients who were symptomatic while on therapy with LT4 (9). The statement recommends that studies should be appropriately powered to study clinical outcomes, the potential interference of gene polymorphisms affecting thyroid hormone signaling, and include patients dissatisfied with their current therapy. Patient preference should be considered as a secondary outcome.

The current investigation involves the prospective crossover study of 75 hypothyroid patients while on 3 different forms of thyroid hormone replacement therapy. In addition to studying outcomes for each treatment arm, we also performed a subanalysis for those patients who were most symptomatic while on the LT4 treatment arm.

## Methods

### Study patients

The trial was widely advertised in various primary care clinics of our hospital. Inclusion criteria included beneficiaries of the military health care system of either sex and between the ages of 18 to 65 years who had been diagnosed with primary hypothyroidism and were on a stable dose of LT4 for at least 6 months. Other criteria included: (1) patient weight of 50 to 100 kg, (2) prestudy LT4 dose of 1.2 to 2.2 mcg/kg/d or a daily LT4 dose of 75 to 250 mcg, and (3) or equivalent dose in terms of combination therapy or DTE (Table 1).

**Table 1.** Baseline patient characteristics

Patient characteristics		(n = 75)
Age, y		50 (range 29-65)
Gender, n (%)		
Female		58 (77.3)
Male		17 (22.7)
Race, n (%)		
Caucasian		58 (77.3)
African American		12 (16.0)
Asian		3 (4.0)
Hispanic		2 (2.7)
Clinical measures		
Weight mean, lb (SD)		180 (40.2)
Cause of hypothyroidism, n (%)		
Autoimmune, Hashimoto		46 (61.3)
Post-radioiodine, Graves’ disease		4 (5.3)
Postthyroidectomy		16 (21.3)
Idiopathic		9 (12.1)
Biochemical measures, mean (SD)		
Total cholesterol	(<200 mg/dL)	199 (39.8)
LDL cholesterol	(<100 mg/dL)	128 (36.0)
HDL cholesterol	(>60 mg/dL)	61.0 (18.6)
Triglyceride	(<150 mg/dL)	106 (60.7)
Total T3	(60-181 ng/dL)	109 (27.3)
T3 resin uptake	(22-35%)	28.0 (3.53)
T3 reverse	(11-32 ng/dL)	19.9 (6.81)
TSH	(0.27-4.20 uIU/mL)	1.77 (1.10)
Total T4	(4.5-12 μg/dL)	8.21 (2.22)
Free T4	(0.89-1.76 ng/dL)	1.41 (0.37)
Free T4 direct dialysis	(0.8-2.7 ng/dL)	1.29 (0.38)
SHBG	(17-124 nmol/L)	71.0 (51.7)
Leptin	(<60 ng/mL for BMI < 30)	20.2 (14.9)
Prestudy medications, mean (SD), (n)		
LT4 dosage (mcg)		112.5 (28.7), (n = 64)
DTE dosage (mg)		71.2 (10.9), (n = 8)
LT4 + LT3 dosage (mcg)		97.3 (16.2) + 5 (0), (n = 3)

Abbreviations: DTE, desiccated thyroid extract; HDL, high density lipoprotein; LDL, low density lipoprotein; LT3, liothyronine; LT4, levothyroxine.

Patients were excluded for the following reasons: pregnancy, plan for pregnancy in the next 12 months, cardiac disease (particularly coronary artery disease), chronic obstructive lung disease, malabsorption disorder, gastrointestinal surgeries, significant renal or liver dysfunction, seizure disorders, thyroid and nonthyroid active cancers, uncontrolled psychosis, and certain medications, including psychotropic medications, corticosteroids, amiodarone, chemotherapy for cancer, iron supplements, sucralfate, proton pump inhibitors, and cholestyramine. Pregnancy was excluded by urine or serum human chorionic gonadotropin test and by taking patient history of missing a menstrual period.

### Study design

The proposed study design was a prospective, randomized, double-blind, crossover study, registered with ClinicalTrials.gov (NCT02317926). After informed consent was obtained, patients were carefully counseled not to make any changes in diets, exercise regimen, or any medications, including lipid-lowering drugs.

The pharmacy department assisted with the preparation, storage, and dispensing of DTE or LT4/LT3 combination or LT4 alone in identical capsules (gelatin capsules, Capsuline Inc., Pompano Beach, FL; Armour Thyroid tablets, USP, Abbvie, Madison NJ; Synthroid, levothyroxine sodium tablet, USP, Abbvie, Madison NJ; Cytomel, Liothyronine tablet, Pfizer Pharmaceuticals, New York, NY).

All study participants, physician investigators, those administering the neurocognitive tests, and those analyzing test results were blinded throughout the study.

Baseline assessments were obtained before randomization, whereas patients were taking either LT4, LT4 + LT3, or DTE (Table 1). Subsequently, patients were randomized to 1 of 3 groups and started on the study medications (Table 2). All medications were given once daily only, in the morning on an empty stomach with water only; other medications, if any, were given at least 1 hour later. Adherence was evaluated through interviews, by counting the number of remaining capsules, and monitoring refills, as well as evaluating the thyroid function tests.

**Table 2.** Equivalent doses of LT4, LT4 + LT3 and DTE given to study subjects

Capsules	1	2	3	4	5	6	7	8	9
LT4 (mcg)	88	100	112	125	137	150	175	200	250
LT4 + LT3 (mcg)	63/7.5	75/7.5	82/7.5	88/10	100/10	112/10	125/12	150/15	175/20
DTE (mg)	60	67.5	75	82.5	90	105	120	135	165

The contents of each one of the 9 capsules are shown; capsules were prepared by the research pharmacy.

Abbreviations: DTE, desiccated thyroid extract; LT3, liothyronine; LT4, levothyroxine.

Each study medication was administered during a study period of 22 weeks, as follows: after the first 6 weeks on the medication, TSH levels were checked, and the dose adjusted to maintain TSH level between 0.27 and 4.20. The adjustment was performed by a physician who had no other contact with the study patients. Adjustments were made by increasing or decreasing the dosage of the study medication as per Table 2. If the TSH was out of range, the next higher or lower dosage was used. Similarly, the physician doing the evaluations did not know the dose of the study medications the patients were taking. With the serum TSH level within the desired range, patients continued on the study medication for an additional 16 weeks, completing the study period. Subsequently, patients were crossed over to the next randomized study medication and followed for another 22-week study period. Predefined equivalence ratios among LT4, LT4 + LT3, and DTE were used (Table 2). The same steps were followed after crossover to the third study medication. The doses of each study medication at the beginning and end of each study period were: LT4, 115 ± 25 vs 115 ± 25 mcg/d; DTE, 77 ± 16 vs 77 ± 17 mg/d; LT4 + LT3, 84 ± 20/8.8 ± 1.7 mcg/d vs 84 ± 20/8.8 ± 1.8 mcg/d (Table 3).

**Table 3.** The doses of study medication at the beginning and end of each study period

Medication	LT4 alone (mcg)	DTE (mg)	LT4 (mcg)/LT3 (mcg)	
Beginning of study period	114.7 ± 25.3	76.7 ± 16.3	83.6 ± 19.7	8.78 ± 1.74
End of study period	115.4 ± 25.1	77.3 ± 16.8	84.1 ± 19.6	8.82 ± 1.75

Abbreviations: DTE, desiccated thyroid extract; LT3, liothyronine; LT4, levothyroxine.

## Assessments

All assessments were performed at baseline and at the end of each study period.

### Clinical assessment

Body weight, resting heart rate, and blood pressure were performed between 07:30 and 09:00 am, while patients were fasting. Also performed were a complete physical examination and a baseline electrocardiogram.

### Clinical biochemistry

Blood samples were collected in the morning between 7 and 10 am, predose in fasting state. Serum TSH, free T4, total T3, and SHBG were measured by clinical diagnostic kits (ECLIA, Cobas 8000, Roche Diagnostics, Indianapolis, IN). Serum lipid panel was measured by homogeneous enzymatic colorimetric methods (Cobas 8000, Roche Diagnostics). Serum total T4 and T3 resin uptake were measured by enzyme immunoassay (AU 5400, Beckman Coulter, TX). Serum free T4 by direct dialysis and reverse T3 were measured by radioimmunoassay (Cal-Biotech, CA, and Radim, Italy, respectively). DNA was extracted from blood samples using manufacturer’s protocol (DNeasy kit, Qiagen). Genotyping was performed in duplicates per allelic discrimination protocol from real-time PCR machine (Applied Biosciences) using TaqMan reagents and rs225014 SNP primer (13,14).

### Questionnaires and cognitive testing

Patients underwent memory testing using the Wechsler memory scale-version IV (WMS-IV) (15,16), Beck Depression Inventory (BDI) (Pearson, PsychCorp, San Antonio, TX) (17,18), and were asked to respond to a QoL general health questionnaire-12 (GHQ-12) (19), and a thyroid symptom questionnaire (TSQ) (17,18). Because there are no well-validated and specific instruments to quantify the severity of hypothyroidism, we designed our own TSQ (12,17), a health-related QoL questionnaire, that was modeled after the hypothyroid-specific questionnaires developed by Jaeschke et al (20) and Cooper et al (21). This questionnaire consisted of 12 questions, presented in the same format as the GHQ-12, that asked patients how they felt over the past 22 weeks. The WMS-IV included 5 subdomains: auditory memory index, visual memory index (VMI), visual working memory index, immediate memory index, and delayed memory index (15,16). The BDI is a self-rating scale of 21 items, in which scores of 10 or less indicate normal mood variation and scores of 11 or more reflect increasing levels of depression; clinically important depression is associated with scores of 20 or more (17,18). At the end of the study, each patient was asked which treatment (the first, the second, or the third) he or she preferred.

### Adverse events

At all visits, patients were specifically asked about side effects, including palpitation, tremor, sweating, and other clinically relevant hypo/hyperthyroid symptoms.

## Outcome Measures

Primary outcome measures included performance in the (1) WMS-IV test, (2) BDI, or (3) TSQ and (4) GHQ-12 questionnaires. Subanalyses included (1) thyroid function tests, (2) body weight and lipid profile, (3) treatment preference, (4) etiology of hypothyroidism, and (5) group subanalysis.

### Statistical analysis

On the basis of a previous study by Clyde et al (17), using the TSQ index as the outcome measure, the means of the 2 groups (LT4 and combination LT4 + T3) were 58 and 50 with the respective standard deviations of 23 and 12. Sample size for this crossover study was based on a paired *t* test with a 5%, 2-sided significance level and assumed a standard deviation of 23 and a within-subjects correlation of 0.5. A sample size of 67 is required for 80% power to detect a difference of 8 points on the TSQ. Accounting for a dropout rate of up to 25%, the necessary sample size is estimated to be 90 enrollees.

#### Primary outcome

Differences between treatments were evaluated using mixed effects models. The primary outcome model included a fixed effect for treatment and a random effect of subject. Models were run with and without the inclusion of baseline scores to isolate between treatment differences. In follow-up analysis of the primary outcome, scores for the LT4 monotherapy condition were also treated as baseline in an adjusted model to isolate effects in the other 2 experimental conditions. The difference between treatments was tested using linear contrasts of the marginal means with Tukey adjustments for between treatment comparisons.

#### Secondary outcomes

Prespecified secondary comparisons included the mixed effects comparison of other collected dependent measures. No multiplicity correction was applied to the large number of these secondary outcome measures.

Additionally, models to evaluate patient preference for each treatment were prespecified. To evaluate these preferences, we used a Plackett-Luce model (22). We also examined the distribution of genotype and clinical indications.

The various dependent measures (including the primary outcome) were tested to identify possible interactions with either patient preference or genotype/clinical characteristics.

#### Subanalysis

Following the logic that various subsets of patients might react differently following treatment, we created terciles of various measures (eg, TSQ, GHQ, BDI,) in the LT4 alone condition and compared patients across those terciles for changes under either DTE or combination LT4/LT3. To evaluate significant differences within those groups, we used the Kruskal-Wallis test and Dunn test for pairwise comparisons, with a Holm adjustment for multiplicity within each measure. One sample exact Wilcoxon tests were used to evaluate whether the change in measures for individual quantiles were different from 0. No omnibus multiplicity correction was applied to these subanalyses, run on a large number of secondary outcome measures.

Categorical comparisons, such as the relationship between gene patterning and diagnosis, were evaluated with χ ^2^ tests or the Fisher exact test, as appropriate. All statistical analyses were completed using R (R Core Team, Vienna, Austria). Alpha was set at 0.05 for all analyses.

## Results

**Figure 1.**
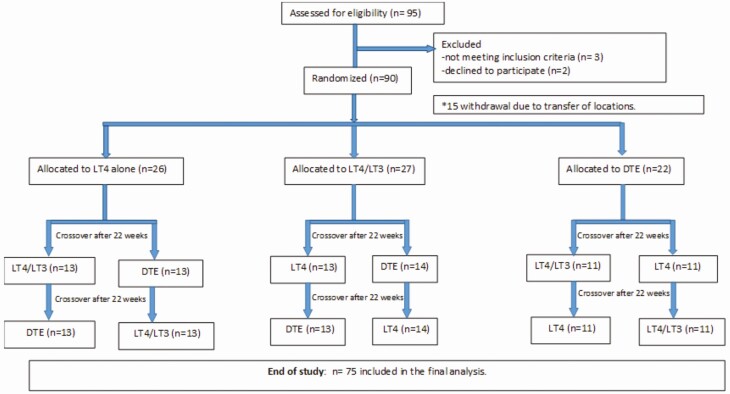
Flow diagram: enrollment, allocation, and completion of the study. Numbers of patients in each arm indicated in parentheses. DTE, desiccated thyroid extract; LT3- liothyronine; LT4, levothyroxine.

Ninety patients were enrolled; 75 patients completed the study. Fifteen patients withdrew from the study before randomization because of relocations and time conflicts (Fig. 1). Of those patients that completed the study, most were Caucasian (77.3%), females (77.3%) averaging 50 years of age (range 29-65 years), and weighing approximately 180 pounds (Table 1). The etiologies of the primary hypothyroidism included autoimmune (46 patients, 61.3%), post-radioactive iodine (4 patients, 5.3%), postthyroidectomy (16 patients, 1 patient with Graves orbitopathy, 21.3%), and idiopathic (9 patients, 12.1%). At the time of the enrollment, most patients (>90%) were on monotherapy with levothyroxine. The thyroid function tests, lipid profile, SHBG, and leptin serum levels were within normal ranges (Table 1).

## Primary Outcome Measures

No differences were observed in the TSQ-36 and GHQ-12 questionnaires, or in the BDI and the 5 subdomains of WMS-IV test assessments (Table 4).

**Table 4.** Primary and secondary outcomes

Parameters	LT4 (N = 75)	DTE (N = 75)	LT4 + LT3 (N = 75)	*P* value
Primary outcomes				
Quality of life and cognition				
TSQ-36	15.9 (7.49)	14.5 (8.22)	14.9 (6.67)	0.357
GHQ-12	11.9 (5.13)	11.7 (6.16)	11.6 (4.83)	0.891
BDI	7.15 (7.11)	7.09 (8.55)	7.33 (7.67)	0.947
AMI	121 (12.8)	121 (14.7)	120 (14.0)	0.825
VMI	80.4 (12.1)	79.8 (8.06)	81.0 (11.3)	0.518
VWMI	116 (14.3)	115 (18.2)	117 (17.2)	0.461
IMI	99.7 (10.7)	97.9 (15.4)	98.0 (15.2)	0.537
DMI	101 (12.0)	101 (10.8)	100 (16.3)	0.827
Secondary outcomes				
Thyroid function tests				
T3 (60-181 ng/dL)	103 (22.1)	155 (48.7)	132 (33.2)	<0.001
T3 resin	28.1 (3.48)	25.8 (4.01)	26.6 (3.95)	<0.001
T3 reverse	20.6 (5.60)	14.4 (5.31)	16.7 (5.02)	<0.001
TSH	1.63 (1.28)	2.34 (1.48)	1.76 (1.13)	<0.001
Total T4	8.46 (2.14)	5.62 (1.38)	6.92 (1.62)	<0.001
Free T4	1.44 (0.358)	0.980 (0.673)	1.21 (0.532)	<0.001
Free T4 direct dialysis	1.30 (0.292)	0.828 (0.293)	1.06 (0.286)	<0.001
Total T4/T3 ratio	82.1(9.7)	36.2(2.8)	52.4 (4.9)	<0.001
Metabolism				
Weight (lbs)	181 (38.7)	180 (40.0)	178 (43.6)	0.278
Total cholesterol	195 (42.5)	196 (37.1)	195 (38.2)	0.85
LDL cholesterol	130 (35.3)	127 (33.6)	126 (35.8)	0.31
HDL cholesterol	59.7 (15.6)	60.5 (18.9)	60.4 (17.8)	0.773
Triglyceride	109 (74.8)	109 (65.7)	102 (63.5)	0.411
SHBG	72.3 (48.1)	69.5 (37.9)	75.0 (45.7)	0.08
Leptin	22.1 (17.9)	21.5 (17.7)	22.2 (18.6)	0.826

*P* value reports the results of a linear mixed effects model evaluating the fixed effect of the 3 treatment conditions, using participant as a random effect.

Abbreviations: AMI, auditory memory index; BDI, Beck Depression Inventory; DTE, desiccated thyroid extract; DMI, delayed memory index; GHQ-12, 12-point quality of life general health questionnaire; HDL, high-density lipoprotein; LDL, low-density lipoprotein; IMI, immediate memory index; LT3, liothyronine; LT4, levothyroxine; TSQ-36, 36-point thyroid symptom questionnaire; VMI, visual memory index; VWMI, visual working memory index.

## Secondary Analyses

Serum TSH remained within the normal refence range and varied minimally among the 3 treatment arms, although levels were slightly higher in DTE-treated patients (Table 4). In contrast, serum T3 and T4 levels were substantially affected by both treatment arms that contained T3:fasting total T3 was 30% to 50% higher and serum T4 levels approximately 30% lower when patients were on therapy with DTE or LT4 + LT3 (Table 4). There was a 38% drop in the total T4/T3 ratio after the patients crossed over to LT4 + LT3, and a 56% drop after they crossed over to DTE. The changes in T4/T3 ratio reflect the changes in serum T4 and T3 levels and remained within the normal reference range (22.7-150). These values, however, are not fully representative of the 24-hour period for the short half-life of T3.

Serum reverse T3, T3 resin, and free T4 were also affected, as expected (Table 4). Body weight, serum lipid levels (ie, total cholesterol, low-density lipoprotein and high-density lipoprotein cholesterol), as well as SHBG and leptin serum levels were not different during the different treatment arms (Table 4). Although blood pressure was not different among the three treatment arms, heart rate was minimally increased while patients were on the DTE treatment arm (Table 5).

**Table 5.** Blood pressure and heart rate during the three treatment arms

	LT4 (N = 75)	DTE (N = 75)	LT4 + LT3 (N = 75)	*P* value
HR				
Mean (SD)	71.5 (11.1)	73.7 (14.3)	70.4 (14.6)	0.04
Diastolic				
Mean (SD)	73.6 (9.24)	74.3 (9.46)	74.3 (8.34)	0.64
Systolic				
Mean (SD)	118 (13.7)	119 (14.5)	121 (14.7)	0.13

*P* value reports the results of a linear mixed effects model evaluating the fixed effect of the 3 treatment conditions, using participant as a random effect.

Abbreviations: DTE, desiccated thyroid extract; LT3, liothyronine; LT4, levothyroxine.

*Treatment preference*

Most patients indicated a treatment preference at the end of the trial (Table 6) but 4 patients had no preference; 2 patients ranked LT4 as the best, and ranked both DTE and LT4/LT3 combination in second place; and 1 patient ranked both DTE and LT4/LT3 as best and LT4 as second. In addition, 1 patient ranked LT4 and DTE as best and ranked LT4/LT3 in second place. There were no significant differences among the 3 treatment groups.

**Table 6.** Treatment preference

Rank	LT4	DTE	LT4 + LT3
1	17 (23%)	34 (45%)	24 (32%)
2	31 (41%)	18 (24%)	25 (33%)
3	22 (29%)	19 (25%)	23 (31%)

Four patients ranked all regimens the same (first for all of them); 2 patients ranked LT4 first and both DTE and LT4/LT3 as second; 1 patient ranked DTE and LT4/LT3 as first and LT4 as second; 1 patient ranked LT4 and DTE as first and LT4/LT3 as second.

Abbreviations: DTE, desiccated thyroid extract; LT3, liothyronine; LT4, levothyroxine.

*Etiology of hypothyroidism and Thr92Ala-DIO2 polymorphism*

No differences in outcomes were identified when patients with autoimmune hypothyroidism vs nonautoimmune disease were compared (Table 7) or when the Thr92Ala-DIO2 polymorphism was considered (Table 8). Of note, the number of subjects with homozygous DIO2 polymorphism was likely too small for meaningful evaluation.

**Table 7.** Primary and secondary outcomes based on etiology of hypothyroidism

Parameters	Autoimmune (N = 153)	Nonautoimmune (N = 72)	*P* value
Primary outcomes			
Quality of life and cognition			
TSQ-36	14.7 (7.39)	15.9 (7.65)	0.414
GHQ-12	11.6 (5.41)	12.2 (5.34)	0.603
BDI	7.21 (8.00)	7.15 (7.29)	0.974
AMI	123 (13.2)	117 (14.3)	0.0657
VMI	80.1 (10.5)	80.9 (10.9)	0.732
VWMI	117 (16.9)	113 (15.8)	0.279
IMI	98.9 (15.2)	97.6 (10.6)	0.617
DMI	101 (14.1)	98.7 (10.9)	0.314
Secondary outcomes			
Thyroid function tests			
T3 (60-181 ng/dL)	129 (41.1)	133 (44.2)	0.43
T3 resin (22%-35%)	26.9 (3.67)	26.8 (4.42)	0.953
T3 reverse (11-32 ng)	17.4 (6.27)	16.8 (5.01)	0.538
TSH (.27-4.68 ulU)	2.11 (1.34)	1.49 (1.22)	*0.0097*
TT4 (4.5-12 mcg/dL	7.15 (2.08)	6.67 (2.09)	0.174
FT4 (.89-1.76 ng)	1.24 (0.655)	1.15 (0.302)	0.481
FT4 direct (.8-2.7 ng/dL)	1.05 (0.326)	1.10 (0.387)	0.313
Metabolism			
Weight (lbs)	177 (41.6)	183 (38.5)	0.536
Tot cholesterol	195 (37.8)	196 (42.1)	0.938
LDL	128 (35.5)	127 (33.3)	0.865
HDL	58.2 (15.2)	64.5 (20.7)	0.117
Triglyceride	109 (70.2)	101 (63.0)	0.586
SHBG (17-124 nmol/L)	70.3 (38.6)	76.6 (53.8)	0.551
Leptin	21.4 (17.4)	23.2 (19.3)	0.673

*P* value reports the results of a linear mixed effects model evaluating the fixed effect of hypothyroidism etiology, adjusted for the 3 treatment conditions, using participant as a random effect.

Abbreviations: AMI, auditory memory index; BDI, Beck Depression Inventory; DTE, desiccated thyroid extract; DMI, delayed memory index; GHQ-12, 12-point quality of life general health questionnaire; HDL, high-density lipoprotein; LDL, low-density lipoprotein; IMI, immediate memory index; LT3, liothyronine; LT4, levothyroxine; TSQ-36, 36-point thyroid symptom questionnaire; VMI, visual memory index; VWMI, visual working memory index.

**Table 8.** Patients’ characteristics presented after the group was broken down by TSQ-36 scores while on therapy with LT4 (L, M, or H)

Parameters	L (N = 30)	M (N = 25)	H (N = 20)	*P* value
Age	49.8 (11.1)	49.7 (8.29)	51.0 (7.64)	0.877
Sex				
Female	24 (80.0%)	18 (72.0%)	16 (80.0%)	0.779
Male	6 (20.0%)	7 (28.0%)	4 (20.0%)	
Genotype_group.y				
Heterozygous	14 (46.7%)	15 (60.0%)	12 (60.0%)	0.679
Ala92-DIO2	3 (10.0%)	3 (12.0%)	3 (15.0%)	
Thr92-DIO2	13 (43.3%)	7 (28.0%)	5 (25.0%)	
Etiology				
Autoimmune	19 (63.3%)	16 (64.0%)	11 (55.0%)	0.795
Nonautoimmune	11 (36.7%)	9 (36.0%)	9 (45.0%)	
Preference				
LT4	12 (40.0%)	1 (4.0%)	2 (10.0%)	0.00892
DTE	9 (30.0%)	13 (52.0%)	7 (35.0%)	
LT4 + LT3	5 (16.7%)	8 (32.0%)	10 (50.0%)	
No preference	4 (13.3%)	3 (12.0%)	1 (5.0%)	
Preference for LT4				
Yes	12 (40.0%)	1 (4.0%)	2 (10.0%)	0.0017
No	18 (60.0%)	24 (96.0%)	18 (90.0%)	
TSH (0.27-4.68 mU/μL)	1.93 (1.31)	1.54 (1.23)	1.32 (1.24)	0.23
T4 (4.5-12 mcg/dL)	8.20 (2.36)	8.77 (1.34)	8.45 (2.60)	0.63
T3 (60-181 ng/dL)	102 (26.9)	103 (14.5)	105 (23.0)	0.896
Reverse T3 (11-32 ng)	19.6 (5.62)	20.3 (4.71)	22.6 (6.35)	0.171

TSQ-36 questionnaire scores presented as low (L), medium (M), and high (H); TSQ-36: L = 1-13, M = >13-21, H = >21-33; for age, serum TSH, T4, T3, and reverse T3 values are mean (SD). For categorical variables, *P* value reports the results of a Fisher exact test; for continuous variables, *P* value reports the results of a linear model evaluating the effect of TSQ score quantile.

Abbreviation: TSQ-36, 36-point thyroid symptom questionnaire.

### Subanalyses

There is now a consensus that a fraction of the LT4-treated hypothyroid patients with normal serum TSH levels remains symptomatic (6,7,23,24). To focus our analyses on symptomatic patients, we first ranked the 75 patients, while they were on LT4, according to their scores on the cognitive tests and questionnaires and stratified them in 3 terciles: low (L), medium (M) and high (H). For TSQ-36, GHQ-12, and BDI, the H group contained the most symptomatic patients, whereas for the WMS-IV (VMI subdomain), the L group contained the patients with the most substantial cognitive impairment. An analysis of the patients’ characteristics in L, M, or H groups did not reveal differences in demographics, Thr92Ala-DIO2 polymorphism, etiology of the disease, or serum thyroid function tests (Table 8). Notably, the treatment preference values mass away from LT4 in the M and H quantiles (Table 8).

**Figure 2.**
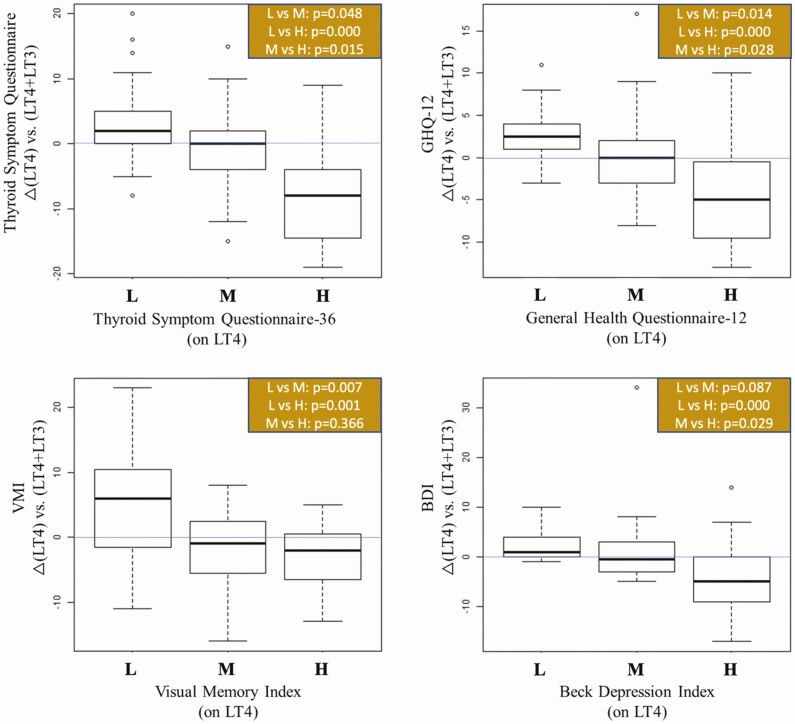
Boxplots showing change scores from LT4 to the LT4/LT3 combination treatment, segmented by either low, medium, or high scores for those measures while on LT4. *P* value reports the results of a pairwise Dunn test for each set of comparisons. Multiple comparisons are adjusted using the Holm method.

**Figure 3.**
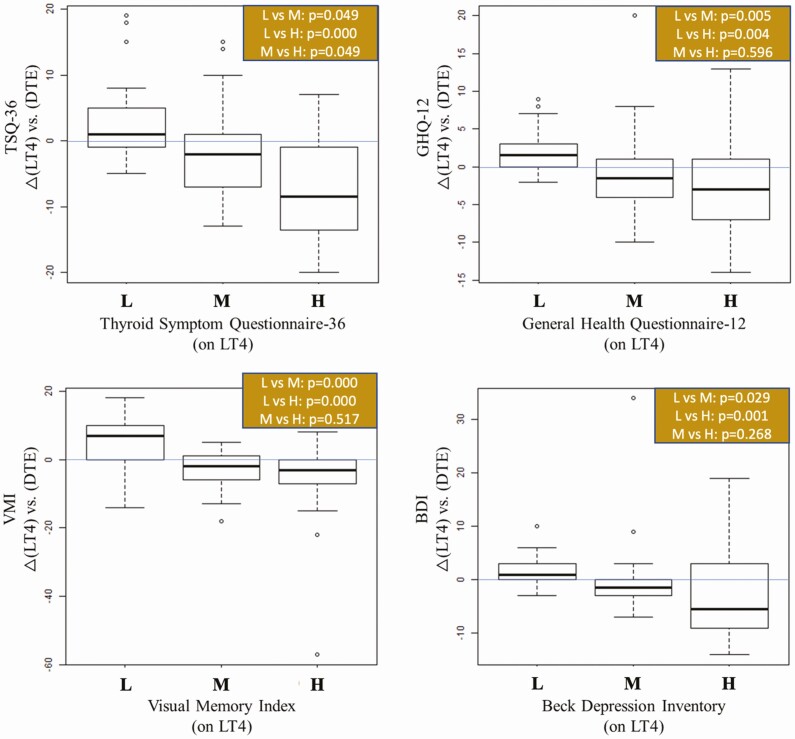
Boxplots showing change scores from LT4 to the DTE treatment, segmented by either low, medium, or high scores for those measures while on LT4. *P* value reports the results of a pairwise Dunn test for each set of comparisons. Multiple comparisons are adjusted using the Holm method.

Next, we asked how patients in each tercile (L, M, or H) performed after they were switched to LT4 + LT3 or DTE, using the strict criterion of *P* < 0.001 to reject the null hypothesis. For the TSQ-36, patients in the H tercile (worst performers on LT4) had statistically different changes than participants in the other L or M terciles, showing substantial improvement after they were switched to LT4 + LT3 (*P* = 0.0005, Fig. 2) or DTE (*P* = 0.0008, Fig. 3).

A similar profile was obtained for BDI (Figs. 2 and 3). The worst performers (tercile H) were those that improved the most from the switch to LT4 + LT3 (Fig. 2) or to DTE (Fig. 3). The pattern was also similar for the GHQ-12, with patients on the L tercile (best performers) being slightly worse after switching to LT4 + LT3, whereas patients on the H tercile (worst performers) did substantially better (Fig. 2).

**Figure 4.**
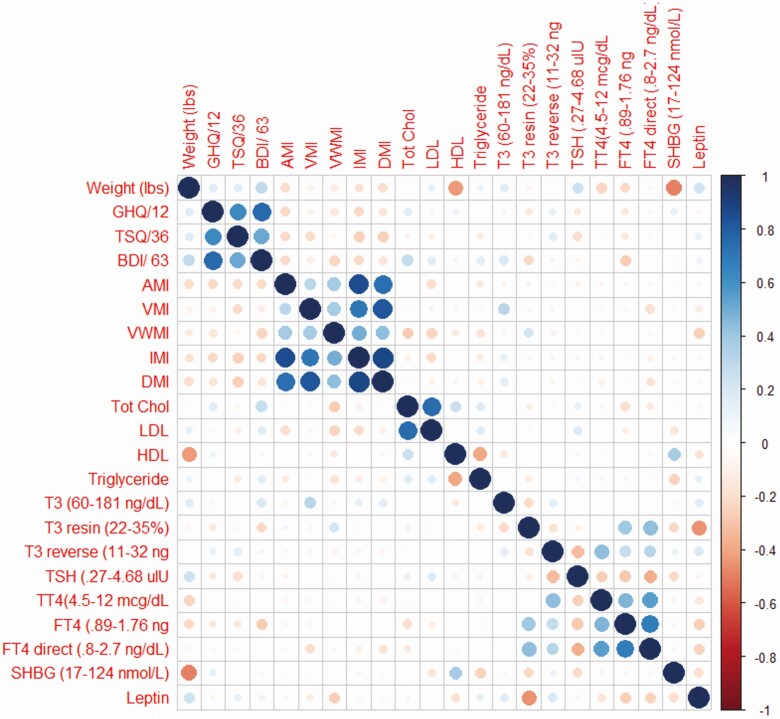
A visualization of the pairwise correlation matrix between the various dependent measures, collected during treatment A. Positive correlations are displayed in blue with negative correlations displayed in red; the size of the circle and the intensity of the color denotes correlation strength. The diagonal is marked with a straight line of solid blue circles.

For the GHQ-12, the switch to DTE had a numerically, but not statistically positive impact on scores in the M and H terciles, improving their relative performance in both cases (Fig. 3). The findings that TSQ-36, GHQ-12, and BDI exhibited similar outcomes were unsurprising given that these tests use similar parameters to assess well-being and QoL, and their scores exhibited a high degree of correlation (Fig. 4).

**Figure 5.**
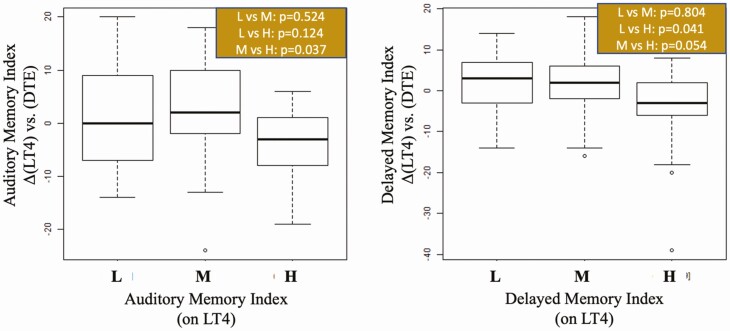
Boxplots showing change scores from LT4 to the DTE treatment, segmented by either low, medium, or high scores for those measures while on LT4. *P* value reports the results of a pairwise Dunn test for each set of comparisons. Multiple comparisons are adjusted using the Holm method.

For the WMS-IV subdomains, only those patients on the L tercile of the VMI (worst performers) improved by switching to LT4 + LT3 (Fig. 2) or DTE (Fig. 3). There was good correlation among the scores in the WMS-IV subdomains (Fig. 4), but only fewer substantial changes were seen with auditory memory index and delayed memory index after patients switched to DTE (Fig. 5). No statistically significant differences in tercile outcomes were observed for visual working memory index and IMI. Likewise, none of the biochemical and metabolic parameters assessed responded favorably to LT4 + LT3 or DTE, even when the L, M, and H terciles were considered.

### Adverse drug reactions

No adverse effects were reported with any of the treatments. All patients tolerated the treatments equally well. None was withdrawn from the study because of side effects.

## Discussion

To our knowledge, this is the first randomized, double-blind, crossover study that compares LT4 vs DTE vs LT4/LT3 therapy in hypothyroid patients. The results confirmed previous trials in which no major differences were observed between monotherapy and combination therapy, including our own previous trial comparing LT4 and DTE (12). Treatment preference was not different and there were no interferences of the etiology of hypothyroidism or Thr92Ala-DIO2 gene polymorphism in the outcomes. However, a subanalysis revealed that the 1/3 most symptomatic patients on LT4-treated hypothyroid patients improved significantly after switching to combination therapy containing T3, either LT4 + LT3, or DTE. They also preferred therapy containing T3 or with DTE. These individuals were identified through poor scores on mood (BDI) and cognitive (VMI) assessments as well as through 2 QoL questionnaires, TSQ-36 and GHQ-12, whereas they were on LT4. Notably, the etiology of the hypothyroidism and the presence of the Thr92Ala-DIO2 polymorphism did not affect these outcomes. These findings are remarkable, as they confirmed anecdotal reports that only a fraction of the LT4-treated hypothyroid patients respond positively to combination therapy containing T3.

Previous studies that compared monotherapy with LT4 and combination therapy containing T3 had mixed results (8,9). This suggests that “responsiveness to therapy containing T3” depends on multiple factors, including genetic background, presence of comorbidities/autoimmune disorders, as well as other yet unidentified biological/environmental factors (25). In general, trials of combination therapy have not been designed to specifically recruit dissatisfied patients on LT4 monotherapy. In fact, some trials have excluded patients with mental illness, affective disorders, or untreated depression (11,26,27). Other trials prescreened and stratified patients for fatigue (28) or depressive symptoms (29). Although we did not specifically recruit symptomatic patients on LT4, here we did use GHQ-12 and TSQ-36, and cognitive dysfunction using WMS-IV evaluation to quantify dissatisfaction and residual symptoms while on LT4 monotherapy. The patients identified through these methods consistently preferred and performed better while on therapy containing T3, either LT4 + LT3, or DTE.

The rationale behind thyroid hormone replacement therapy is to administer thyroid hormone in a way that restores thyroid hormone signaling in all tissues. Serum TSH has traditionally been used to adjust the dose of levothyroxine that presumably achieves this goal. However, normalization of serum TSH is achieved through a slightly elevated serum T4 and reduced serum T3 levels (25). TSH was higher in the DTE arm but still within the normal range. It was difficult to make DTE and LT4 doses bioequivalent. It is not known whether these small changes in serum T3 and T4 modify thyroid hormone signaling. The observation in the present investigation that replacement therapy containing T3 or DTE (which also contains T3) slightly elevates serum T3 and reduces serum T4, whereas mitigating the residual symptoms of hypothyroidism suggests a causal relationship. Nonetheless, our findings are clear that no correlation between serum T3 and outcomes could be established in the present investigation, not even in the subanalyses. This apparent conundrum suggests that intracellular T3 levels, which are affected by both serum T3 and locally generated T3, might be playing a role (30).

It is well known that thyroid hormone plays a role in the development of an adult brain, as documented by the modifications in mood, behavior, and cognition observed during the transition from hypo- to euthyroidism and to hyperthyroidism (31,32). In fact, the human temporal pole responds promptly to minimal changes in thyroid hormone signaling (33). Unique about the brain is that thyroid hormone signaling exhibits additional layers of complexity; thus, there are multiple mechanisms that can fail and compromise thyroid hormone signaling. Although plasma T3 can be taken up by the brain, most T3 molecules in the brain are produced locally in the glial cells through deiodination of T4 via DIO2. T3 exits the glial cells and subsequently functions in a paracrine fashion to activate neuronal gene expression (34). That some patients on LT4 monotherapy remain symptomatic and prefer therapy containing T3 suggests that therapy with LT4 might not restore cerebral thyroid hormone in all patients. Hypothetically, this could be explained by defects in thyroid hormone transporters, the DIO2 pathway, or by the relatively low serum T3 levels seen in LT4-treated patients (25), but none of these factors segregated in the most symptomatic LT4-treated patients. The possibility that the relatively higher plasma T4 levels seen in LT4-treated patients might play a role remains to be investigated.

Thus, the simplest way to interpret the present results is to consider that thyroid hormone signaling in the brain was not fully restored in some LT4-treated patients as a result of one or more mechanisms yet to be identified, and that the elevation in serum T3 resulting from treatment with LT4 + LT3 or DTE mitigated this problem. Importantly, despite the increase in serum T3 levels seen with LT4 + LT3 or DTE, there were no associated cardiovascular adverse reactions or changes in blood pressure; heart rate was only minimally accelerated by therapy with DTE. This is in agreement with findings of other studies in which patients treated with liothyronine or DTE were analyzed (3).

In the subanalyses, we did not use baseline values, but rather values on LT4. So, data obtained at the end of DTE or LT4 + LT3 study periods were compared to data obtained at the end of the LT4 study period. While “regression to the mean” may emerge as a possible explanation, we do not believe this is happening because it is unlikely that most cognitive tests would follow the same tendency randomly; in addition, these very same patients were the ones that preferred DTE or combination therapy.

This study was powered to detect a within-subjects difference in TSQ scores between treatments for hypothyroidism, and therefore recruited and studied a group of 75 hypothyroid patients. Thus, a significant limitation of the present investigation was that the most exciting findings were obtained through a subanalysis that was not included in the primary outcomes of the study, and that used a smaller subset of patients to conduct a between-subjects analysis. Again, it is conceivable that regression to the mean explains some of the observed effects; patients that tended to do poorly on the LT4 treatment showed larger improvements when switched to other treatments. However, several observations may ameliorate that possibility. First, the parameters we identified as being responsive to therapy containing T3 (ie, QoL questionnaires and cognitive tests) exhibited intrinsic consistency, and thus are unlikely to reflect random findings. Second, patients who did poorly on LT4 showed, generally, much less preference for LT4, indicative of at least some participant knowledge of the efficacy of the treatment, which may indicate that these groupings are more than simply a statistical artifact. Third, patients who did poorly on LT4 did not necessarily show consistent improvement when switched to either of the other 2 treatments, suggesting that mean reversion does not completely account for the finding. Future studies designed to primarily study symptomatic patients on LT4 should expand and clarify these findings, with a design specifically powered to address the between-subject difference that arises from the idiosyncratic success of patient responsiveness to different therapies.

In conclusion, some patients on LT4 therapy remain symptomatic despite normal serum TSH levels. In the present investigation, the number of these patients was not sufficiently large to affect the outcomes of the whole group. However, in the subset analysis, these patients were identified as having the worst performance on QoL questionnaires and cognitive tests. These patients were also the ones that preferred and benefitted from switching to therapy containing T3, either DTE or LT4 + LT3 combination, suggesting that thyroid hormone signaling in a minority of the patients on LT4 remain subnormal and can be improved (perhaps restored) with therapy containing T3.

## Data Availability

Some or all data generated or analyzed during this study are included in this published article or in the data repositories listed in References.

## Abbreviations

BDI: Beck Depression Inventory
DTE: desiccated thyroid extract
GHQ-12: 12-point quality of life general health questionnaire
H: high
L: low
LT3: liothyronine
LT4: levothyroxine
M: medium
QoL: quality of life
TSQ-36: 36-point thyroid symptom questionnaire
VMI: visual memory index
WMS-IV: Wechsler memory scale-version IV

## Additional Information

**Disclosure Statement:** AB is a consultant for Synthonics, BLA Technology, and Allergan; the other authors declare that no competing financial interests exist.

We certify that all individuals who qualify as authors have been listed; that each has participated in the conception and design of this work, the analysis of data, the writing of the document, and the approval of the submission of this version; that the document represents valid work; that if we used information derived from another source, we obtained all necessary approvals to use it and made appropriate acknowledgements in the document; and that each takes public responsibility for it.

## References

[1] Chaker L , Bianco AC, Jonklaas J, Peeters RP. Hypothyroidism. Lancet. 2017;390(10101):1550-1562. 28336049 10.1016/S0140-6736(17)30703-1 PMC6619426

[2] Aoki Y , Belin RM, Clickner R, Jeffries R, Phillips L, Mahaffey KR. Serum TSH and total T4 in the United States population and their association with participant characteristics: National Health and Nutrition Examination Survey (NHANES 1999-2002). Thyroid. 2007;17(12):1211-1223. 18177256 10.1089/thy.2006.0235

[3] Idrees T , Palmer S, Maciel RMB, Bianco AC. Liothyronine and desiccated thyroid extract in the treatment of hypothyroidism. Thyroid. 2020;30(10):1399-1413. 32279609 10.1089/thy.2020.0153 PMC7640752

[4] Taylor S , Kapur M, Adie R. Combined thyroxine and triiodothyronine for thyroid replacement therapy. Br Med J. 1970;2(5704):270-271. 5420176 10.1136/bmj.2.5704.270 PMC1700438

[5] Roberts ND . Psychological problems in thyroid disease. Br Thyroid Foundation Newsl. 1996;18:3.

[6] Saravanan P , Chau WF, Roberts N, Vedhara K, Greenwood R, Dayan CM. Psychological well-being in patients on ‘adequate’ doses of l-thyroxine: results of a large, controlled community-based questionnaire study. Clin Endocrinol (Oxf). 2002;57(5):577-585. 12390330 10.1046/j.1365-2265.2002.01654.x

[7] Wekking EM , Appelhof BC, Fliers E, et al. Cognitive functioning and well-being in euthyroid patients on thyroxine replacement therapy for primary hypothyroidism. Eur J Endocrinol. 2005;153(6):747-753. 16322379 10.1530/eje.1.02025

[8] Jonklaas J , Bianco AC, Bauer AJ, et al; American Thyroid Association Task Force on Thyroid Hormone Replacement. Guidelines for the treatment of hypothyroidism: prepared by the American Thyroid Association task force on thyroid hormone replacement. Thyroid. 2014;24(12):1670-1751. 25266247 10.1089/thy.2014.0028 PMC4267409

[9] Jonklaas J , Bianco AC, Cappola AR, et al. Evidence-based use of levothyroxine/liothyronine combinations in treating hypothyroidism: a consensus document. Thyroid. 2021;31(2):156-182. 33276704 10.1089/thy.2020.0720 PMC8035928

[10] Appelhof BC , Fliers E, Wekking EM, et al. Combined therapy with levothyroxine and liothyronine in two ratios, compared with levothyroxine monotherapy in primary hypothyroidism: a double-blind, randomized, controlled clinical trial. J Clin Endocrinol Metab. 2005;90(5):2666-2674. 15705921 10.1210/jc.2004-2111

[11] Escobar-Morreale HF , Botella-Carretero JI, Gómez-Bueno M, Galán JM, Barrios V, Sancho J. Thyroid hormone replacement therapy in primary hypothyroidism: a randomized trial comparing L-thyroxine plus liothyronine with L-thyroxine alone. Ann Intern Med. 2005;142(6):412-424. 15767619 10.7326/0003-4819-142-6-200503150-00007

[12] Hoang TD , Olsen CH, Mai VQ, Clyde PW, Shakir MK. Desiccated thyroid extract compared with levothyroxine in the treatment of hypothyroidism: a randomized, double-blind, crossover study. J Clin Endocrinol Metab. 2013;98(5):1982-1990. 23539727 10.1210/jc.2012-4107

[13] Peeters RP , van Toor H, Klootwijk W, et al. Polymorphisms in thyroid hormone pathway genes are associated with plasma TSH and iodothyronine levels in healthy subjects. J Clin Endocrinol Metab. 2003;88(6):2880-2888. 12788902 10.1210/jc.2002-021592

[14] Mentuccia D , Proietti-Pannunzi L, Tanner K, et al. Association between a novel variant of the human type 2 deiodinase gene Thr92Ala and insulin resistance: evidence of interaction with the Trp64Arg variant of the beta-3-adrenergic receptor. Diabetes. 2002;51(3):880-883. 11872697 10.2337/diabetes.51.3.880

[15] Wechsler DA. The Wechsler Memory Scalle-IV. 4th ed. San Antonio, TX: Psychological Corp; 2009.

[16] Benson N , Hulac DM, Kranzler JH. Independent examination of the Wechsler Adult Intelligence Scale-Fourth Edition (WAIS-IV): what does the WAIS-IV measure? Psychol Assess. 2010;22(1):121-130. 20230158 10.1037/a0017767

[17] Clyde PW , Harari AE, Getka EJ, Shakir KM. Combined levothyroxine plus liothyronine compared with levothyroxine alone in primary hypothyroidism: a randomized controlled trial. Jama. 2003;290(22):2952-2958. 14665656 10.1001/jama.290.22.2952

[18] Beck AT , Ward CH, Mendelson M, Mock J, Erbaugh J. An inventory for measuring depression. Arch Gen Psychiatry. 1961;4:561-571. 13688369 10.1001/archpsyc.1961.01710120031004

[19] McDowell I , Newell C. The General Health Questionnaire, Measuring Health. A guide to Rating Scales and Questionnaires. 2nd ed. New York, NY: Oxford University Press; 1996.

[20] Jaeschke R , Guyatt G, Cook D, Harper S, Gerstein HC. Spectrum of quality of life impairment in hypothyroidism. Qual Life Res. 1994;3(5):323-327. 7841966 10.1007/BF00451724

[21] Cooper DS , Halpern R, Wood LC, Levin AA, Ridgway EC. L-Thyroxine therapy in subclinical hypothyroidism. A double-blind, placebo-controlled trial. Ann Intern Med. 1984;101(1):18-24. 6428290 10.7326/0003-4819-101-1-18

[22] Turner HL , van Etten J, Firth D, Kosmids J. Modelling rankings in R: the PlackettLuce package. Comput Stat. 2020;35:1027-1057.

[23] Peterson SJ , Cappola AR, Castro MR, et al. An online survey of hypothyroid patients demonstrates prominent dissatisfaction. Thyroid. 2018;28(6):707-721. 29620972 10.1089/thy.2017.0681 PMC6916129

[24] Peterson SJ , McAninch EA, Bianco AC. Is a normal TSH synonymous with “euthyroidism” in levothyroxine monotherapy? J Clin Endocrinol Metab. 2016;101(12):4964-4973. 27700539 10.1210/jc.2016-2660 PMC6287526

[25] Ettleson MD , Bianco AC. Individualized therapy for hypothyroidism: is T4 enough for everyone? J Clin Endocrinol Metab. 2020;105(9):e3090-e3104. 10.1210/clinem/dgaa430 PMC7382053 32614450

[26] Valizadeh M , Seyyed-Majidi MR, Hajibeigloo H, Momtazi S, Musavinasab N, Hayatbakhsh MR. Efficacy of combined levothyroxine and liothyronine as compared with levothyroxine monotherapy in primary hypothyroidism: a randomized controlled trial. Endocr Res. 2009;34(3):80-89. 19701833 10.1080/07435800903156340

[27] Walsh JP , Shiels L, Lim EM, et al. Combined thyroxine/liothyronine treatment does not improve well-being, quality of life, or cognitive function compared to thyroxine alone: a randomized controlled trial in patients with primary hypothyroidism. J Clin Endocrinol Metab. 2003;88(10):4543-4550. 14557419 10.1210/jc.2003-030249

[28] Rodriguez T , Lavis VR, Meininger JC, Kapadia AS, Stafford LF. Substitution of liothyronine at a 1:5 ratio for a portion of levothyroxine: effect on fatigue, symptoms of depression, and working memory versus treatment with levothyroxine alone. Endocr Pract. 2005;11(4):223-233. 16006298 10.4158/EP.11.4.223 PMC1455482

[29] Sawka AM , Gerstein HC, Marriott MJ, MacQueen GM, Joffe RT. Does a combination regimen of thyroxine (T4) and 3,5,3’-triiodothyronine improve depressive symptoms better than T4 alone in patients with hypothyroidism? Results of a double-blind, randomized, controlled trial. J Clin Endocrinol Metab. 2003;88(10):4551-4555. 14557420 10.1210/jc.2003-030139

[30] Bianco AC , Dumitrescu A, Gereben B, et al. Paradigms of dynamic control of thyroid hormone signaling. Endocr Rev. 2019;40(4):1000-1047. 31033998 10.1210/er.2018-00275 PMC6596318

[31] López-Espíndola D , García-Aldea Á, Gómez de la Riva I, et al. Thyroid hormone availability in the human fetal brain: novel entry pathways and role of radial glia. Brain Struct Funct. 2019;224(6):2103-2119. 31165302 10.1007/s00429-019-01896-8

[32] Morte B , Bernal J. Thyroid hormone action: astrocyte-neuron communication. Front Endocrinol (Lausanne). 2014;5:82. 24910631 10.3389/fendo.2014.00082 PMC4038973

[33] Marcelino CP , McAninch EA, Fernandes GW, Bocco BMLC, Ribeiro MO, Bianco AC. Temporal pole responds to subtle changes in local thyroid hormone signaling. J Endocr Soc. 2020;4(11):bvaa136. 33123655 10.1210/jendso/bvaa136 PMC7575126

[34] Freitas BC , Gereben B, Castillo M, et al. Paracrine signaling by glial cell-derived triiodothyronine activates neuronal gene expression in the rodent brain and human cells. J Clin Invest. 2010;120(6):2206-2217. 20458138 10.1172/JCI41977 PMC2877954

